# The Effect of Fatalistic Tendency in Individuals on Attitudes Toward Epilepsy Patients

**DOI:** 10.1002/brb3.70160

**Published:** 2025-01-07

**Authors:** Öznur Adadioğlu, Ahmet Seven, Metin Yıldız

**Affiliations:** ^1^ Department of Nursing Sakarya University Sakarya Türkiye; ^2^ Department of Nursing, Afsin Faculty of Health Sciences Kahramanmaraş Sütçü İmam University Kahramanmaraş Türkiye

**Keywords:** attitudes, epilepsy, fatalism tendency

## Abstract

**Objective:**

This study was conducted to determine the effect of fatalistic tendency on attitudes toward epilepsy patients.

**Methods:**

The study was conducted between August 17 and October 1, 2022 in a family health center in Sakarya province in western Türkiye. The sample consisted of 479 adults. Data were collected in descriptive information forms, the fatalism tendency scale and the epilepsy attitude scale. The data were analyzed using SPSS 22.0, AMOS V 24.0, and G*Power 3.1 statistical package programs.

**Results:**

Results showed that the model built according to the hypotheses was compatible, and the model fit indices *χ* ^2^/Sd = 1.857, RMSEA = 0.04, CFI = 0.98, GFI = 0.99, AGFI = 0.97, and IFI = 0.98 were within the desired limits. Structural equation modeling to determine the effect of fatalistic tendency on attitudes toward epilepsy revealed that fatalistic tendency affected the attitudes toward epilepsy patients (*β* = 0.87, *p* < 0.05).

**Conclusion:**

In our study, as the fatalistic tendency of individuals increased, their negative attitudes toward epilepsy patients increased. Longitudinal studies on attitudes toward epilepsy patients are recommended.

## Graphical abstract

In this study, Figures 1 and 2 show the confirmatory factor analysis of the scale and Figure 3 shows the structural equation modeling validation. Confirmatory factor analysis of the fatalism tendency scale is shown in Figure 1. Confirmatory factor analysis indicated satisfactory fit indices: *χ* ^2^/df = 2.238, RMSEA = 0.05, CFI = 0.90, GFI = 0.91, AGFI = 0.89, and IFI = 0.90, confirming the structural integrity of the scale (Figure 1). Confirmatory factor analysis of the public attitudes toward epilepsy scale is shown in Figure 1. Confirmatory factor results of the analysis, the goodness of fit indices were *χ* ^2^/Sd = 3.777, RMSEA = 0.07, CFI = 0.93, GFI = 0.91, AGFI = 0.88, IFI = 0.93, and TLI = 0.91, and the structure of the scale was confirmed (Figure 2). When the results of the structural equation model were analyzed, the compatibility of the model was confirmed in line with the hypotheses, and the model fit indices demonstrated satisfactory results: χ^2^/df = 1.857, RMSEA = 0.04, CFI = 0.98, GFI = 0.99, AGFI = 0.97, and IFI = 0.98. These indices indicated that the model met the desired criteria for fit (Figure 3).

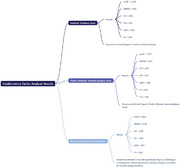

## 1 Introduction

Fatalism is defined as a doctrine that holds that events are predetermined and therefore humans are powerless to change them (De Los Monteros 2020). Although the word fatalism is generally used to express resignation in the face of a future event or events that are considered inevitable, philosophers often use fatalism to refer to our inability to do anything else (Morgan, Tyler, and Fogel 2008). Fatalism is mostly discussed in three ways (Bernstein 1990). Logical fatalism regarding logical laws and rules of metaphysics, theological fatalism regarding the existence of God, and types of fatalism in general in relation to your causal determinism (Haack 1974; Finch, and Warfield 1999; Hunt 2020; Finch 2023).

The concept of fatalism is generally associated with religion in the literature and is more common in individuals with a belief in one God (Kaya and Bozkur 2015; Kıyak et al. 2021). Based on fatalism, according to religious beliefs, God predetermines what will happen to people, what they will experience, fate, and the view that these situations cannot be changed (Kaya and Bozkur 2017; Dayapoğlu, Ayyıldız, and Şeker 2021). Within the scope of the concept of fatalism, it is seen that no matter what we do against the events, we cannot go beyond the existing determination and that there is submission and questioning in all conditions (Kaya and Bozkur 2017).

In a study conducted in Türkiye as an international comparison, it was observed that fatalism is effective on almost one out of every two people in Türkiye compared to other countries (Çarkoğlu and Kalaycıoğlu 2009). Although the fatalistic tendency is effective in all areas of life, it is also effective in the perception of one's health status, especially in the area of health (Pehlivan and Aktas 2022; Özer and Turan 2023). Individuals with a fatalistic tendency believe that their health status is determined by the influence of God or supernatural forces beyond their control (Turan, Dayapoğlu, and Özer 2022). In parallel with this idea, it is seen that negative behaviors such as the occurrence of diseases, having chronic diseases, and not taking precautions in the treatment and care of diseases are more common among people with fatalistic tendencies (Pehlivan and Aktas 2022; Bachem et al. 2020).

Epilepsy is a persistent neurological condition marked by repetitive seizures linked to abnormal and excessive neuronal activity in the brain. This phenomenon manifests across various age groups and genders globally, including within our country (Thijs et al. 2019; Diby et al. 2021). Although physical dysfunction in epilepsy is minimal, some individuals with epilepsy may feel severely disabled. This may be due to the fear of seizures in individuals with epilepsy. They avoid social activities because they are afraid of having a seizure. A negative social environment supports individuals with epilepsy to feel disabled (Rhodes et al. 2008). At the same time, individuals with epilepsy face certain active discrimination, such as bans on obtaining a driver's license and working in certain lines of work, and experience difficulties in accessing health, social, and legal care and assistance (Agudelo‐Hernández, Toro, and Plata‐Casas 2024). Misinformation surrounding epilepsy, a prevalent chronic condition, has resulted in the emergence of negative attitudes and discriminatory behaviors toward individuals affected by the disorder (Heersink et al. 2015; Baker, Eccles, and Caswell 2018).

Seizures in people with epilepsy are associated with fate by some, while others may see them as punishment (Aydemir, Unsal, and Ozkara 2011). In developing countries, social exclusion and negative attitudes are shown because the disease is believed to be contagious under the influence of supernatural forces (Demirel and Okçin 2020). In developed countries, stigma and negative attitudes toward people with epilepsy are caused, especially due to the psychosocial consequences caused by the visually frightening seizures associated with the disease (Baker, Eccles, and Caswell 2018; Şahin, Parlak, and Aydin 2022). In a meta‐analysis study examining the stigmatization experiences of epilepsy patients, it is stated that they experience stigmatization in schools, health clinics, the workplace, and other aspects of social life (in different social relationships, including interaction with family members, friends, neighbors, and lovers) (Shi et al. 2021).

Misconceptions publicly known about epilepsy may cause stigmatization of individuals with epilepsy. In the systematic review conducted by Herrmann et al. (2016), misunderstandings that caused the stigmatization of epilepsy patients were identified. Themes of misunderstanding include limitations in social roles (marriage, children, and employment for individuals with epilepsy), conditions associated with epilepsy (cognitive and behavioral restrictions), seeing individuals with epilepsy as dangerous, restrictions on activities in daily life (sports activities and driving), etiology of epilepsy, and treatment of epilepsy. There are misconceptions about its prognosis (Herrmann et al. 2016). There is a limited amount of research on the effect of fatalistic tendency on attitudes toward epilepsy patients.

Hence, this research is anticipated to make a valuable contribution to the existing literature and provide insights that can illuminate other related studies. The primary objective of this study was to investigate the effect of fatalistic tendencies in adults on attitudes toward epilepsy patients.

With this study, the effect of fatalistic tendency on attitudes toward epilepsy patients was revealed and supported by structural equation modeling (SEM).

Hypotheses of the study are:

**H_0_**. *Fatalistic tendency has no effect on attitudes toward epilepsy. Fatalistic tendency has no effect on attitudes toward epilepsy patients*.
**H_1_**. *Fatalistic tendency has an effect on attitudes toward epilepsy patients*.

### 1.1 Main Points

- There are many factors affecting the attitudes toward epilepsy patients.
- One of the factors affecting the attitudes toward epilepsy patients is fatalism.
- In this study, it was determined at which level and in which direction fatalism affects the attitudes toward epilepsy patients.

## 2 Methods

The population of the study consists of individuals enrolled in a family health center in Sakarya province in western Türkiye. This descriptive and cross‐sectional study was conducted between August 17 and October 1, 2022 in a family health center in Sakarya province in western Türkiye.

The determination of the minimum sample size for the study was computed to be at least 384 individuals, utilizing the sampling formula. A total of 479 individuals were included in the study. Individuals who were 18 years of age or older, had no speech–hearing problems, were cognitively competent (without any mental disability and did not have any psychiatric disorder), and did not have epilepsy who agreed to participate in the study were included. Individuals who applied to the family health center for any reason, such as examination, vaccination, consultation, or injection, were invited to the study. Information was given about the research. The research was conducted with individuals who met the inclusion criteria and volunteered.

Following a post hoc power analysis based on the results obtained from the 479 participants, it was determined that the study achieved a power of 99% at the 95% confidence level, considering a medium effect size (Cohen 1988). The reporting of this research with STROBE guidelines (Vandenbroucke 2007).

### 2.1 Data Collection Tools

The collection of data for this study involved the utilization of a descriptive information form encompassing sociodemographic characteristics, alongside the inclusion of the fatalism tendency scale (FTS) and the Public Attitudes Toward Epilepsy Scale. The data collection process was conducted through face‐to‐face interactions.

#### 2.1.1 Introductory Information Form

This form includes seven questions about the sociodemographic characteristics of the participants.

##### 2.1.1.1 Fatalism Tendency Scale

**FIGURE 1:**
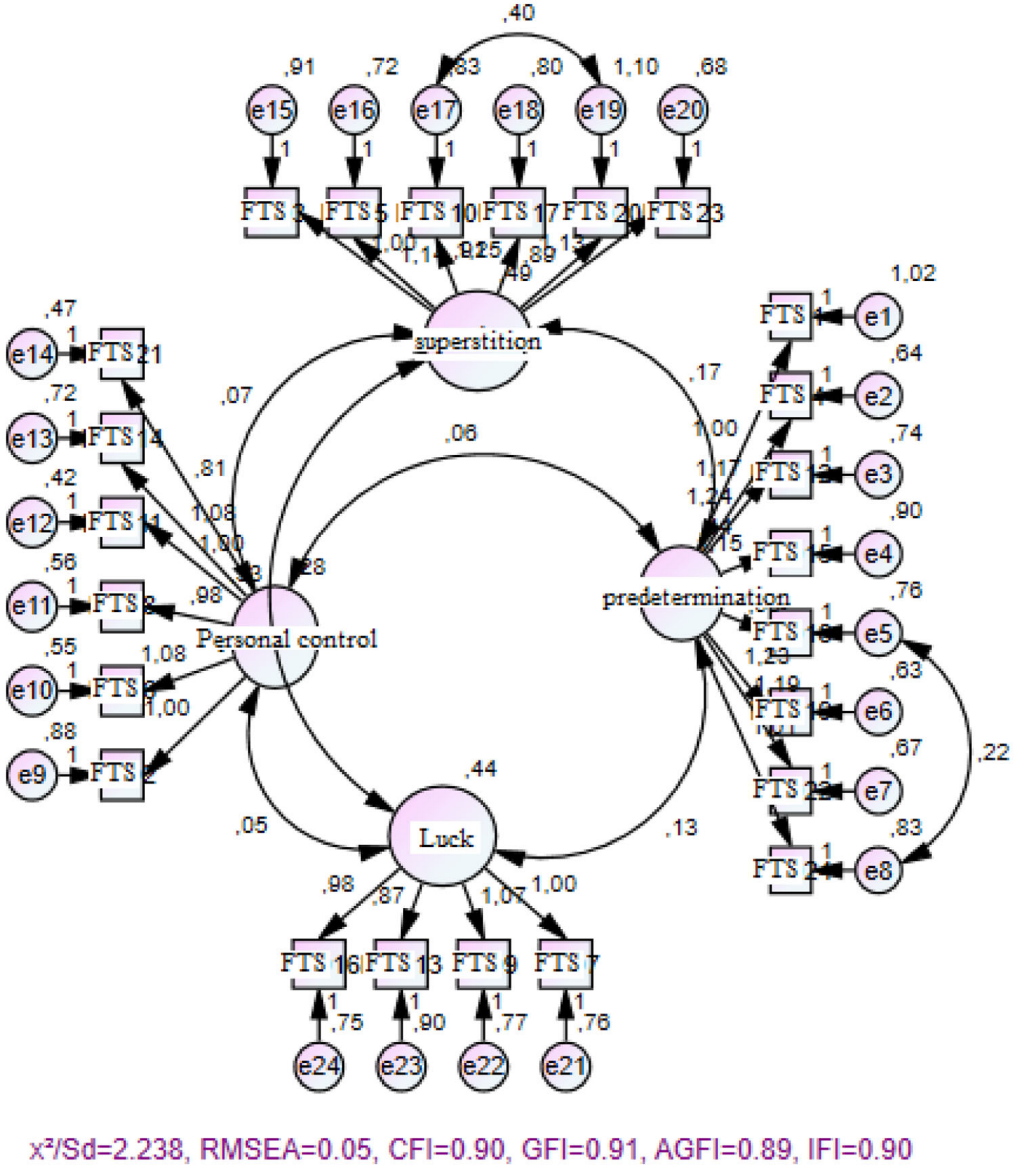
Fatalism tendency scale.

The scale developed by Kaya and Bozkur (2015) measures the fatalistic tendency of individuals. The scale consists of 24 items and 4 subdimensions. The subdimensions of the scale are predestination, personal control, superstition, and luck. The scale is a five‐point Likert‐type scale. In the scale, the items that make up the personal control dimension (2, 6, 8, 11, 14, and 21) are scored in reverse order, while the other three subdimensions are scored directly. The total score ranges from 24 to 120. An increase in score indicates an increase in fatalistic tendency. In the scale by Kaya and Bozkur (2015), Cronbach's alpha was calculated as 0.86 whereas in our work, Cronbach's alpha was calculated as 0.84. Confirmatory factor analysis indicated satisfactory fit indices were *χ* ^2^/df = 2.238, RMSEA = 0.05, CFI = 0.90, GFI = 0.91, AGFI = 0.89, IFI = 0.90 (see Figure 1), confirming the structural integrity of the scale (Karagöz 2019).

##### 2.1.1.2 Public Attitudes Toward Epilepsy Scale

**FIGURE 2:**
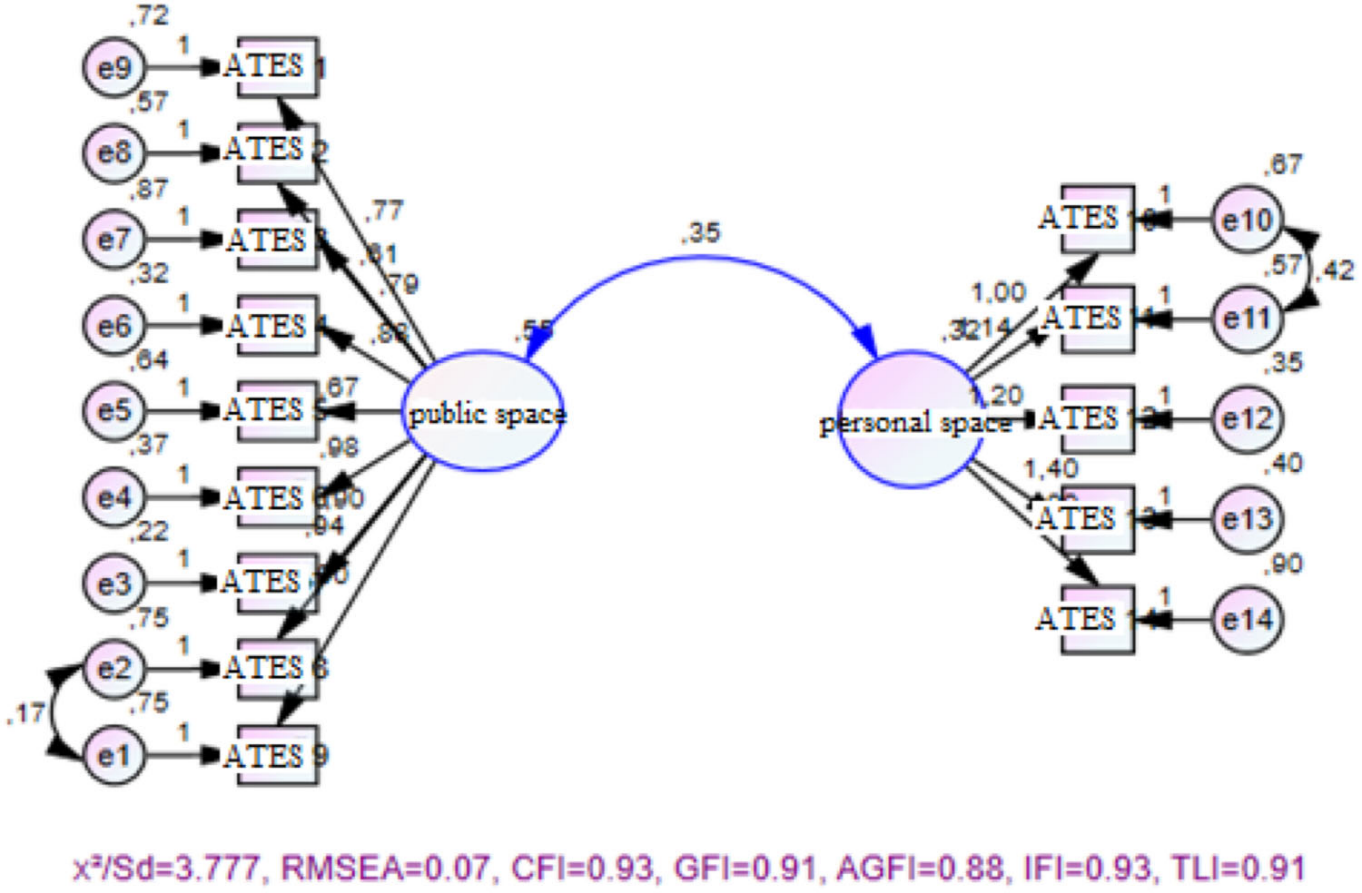
Public attitudes toward epilepsy scale.

The Turkish validity and reliability of the public attitudes toward epilepsy (PATE) scale developed by Lim et al. (2012) was conducted by Aktürk et al. (2020). The scale, which has two subdimensions: general and personal domain, consists of 14 items. The scale is a five‐point Likert‐type scale. The highest score obtained from the scale is 70, and the lowest score is 14. A high score on the scale indicates a negative attitude. Cronbach's alpha was calculated as 0.783 (Aktürk et al. 2020). The Cronbach's alpha value of the current study was 0.895. Confirmatory factor analysis indicated the goodness of fit indices were *χ* ^2^/Sd = 3.777, RMSEA = 0.07, CFI = 0.93, GFI = 0.91, AGFI = 0.88, IFI = 0.93, and TLI = 0.91, and the structure of the scale was confirmed (Figure 2).

### 2.2 Statistical Analysis

The data were analyzed using SPSS 22.0, AMOS V 24.0, and G*Power 3.1 statistical package programs. In the analysis of the study, descriptive statistical analyses were performed with the SPSS package program. Confirmatory factor analysis and structural equation model analysis of the scales were performed with AMOS program. The significance level (*p*) for statistical tests was set at 0.05. The tests used to evaluate the data are listed in Table 1.

**TABLE 1:** Statistical methods used in data analysis.

Assessed characteristics	Statistical methods
Determining the suitability of the data for normal distribution	Skewness coefficient Kurtosis coefficient
Determination of descriptive characteristics	Percentage distribution Frequency distribution
Determining the relationships between variables and creating a model	Structural equation model (Maximum likelihood estimation)
Evaluation of model fit	Fit indices Adjusted chi‐square statistic (*χ* ^2^/Sd) Goodness of fit index (GFI) Adjusted goodness of fit index (AGFI) Comparative fit index (CFI) Root mean square error of approximation (RMSEA) Incremental fit index (IFI)
Model assumption analysis	Multiple normal distribution Skewness value Kurtosis value Distance Mahalanobis Multicollinearity Variance inflation factor (VIF)
Ensuring the validity of measurement tools	Confirmatory factor analysis (Fit indexes)
Ensuring the reliability of measurement tools	Cronbach's alpha coefficient

### 2.3 Structural Equation Modeling

#### 2.3.1 Assumption Analysis

SEM is a multivariate statistical method that provides the ability to test multiple relationships simultaneously and calculates the causal relationship between variables through modeling. All necessary conditions for SEM were met (Gürbüz 2019; Collier 2020; O'Rourke and Hatcher 2013; Yoon, Kim, and Kim 2021; Lee and Lee 2022; Mottaghi, Poursheikhali, and Shameli 2020).

##### 2.3.1.1 Reliability Analyses of the Scales

All necessary reliability was ensured for SEM (Karagöz 2019; Hu and Bentler 1999).

### 2.4 Ethical Approval

Ethical approval, granted with Scientific Ethics Committee Approval No. 2022/88 on April 4, 2022, was obtained from the Ethics Committee of a university. Written informed consent was obtained from the individuals in the study. The research adhered to the principles outlined in the Helsinki Declaration of Human Rights.

## 3 Results

It was found that 62.6% of the individuals who participated in the study were female, 50.5% were single, 37.2% had a higher education degree, 66.2% had an income equivalent to their expenses, 82.5% did not have chronic diseases, 68.9% had information about epilepsy, and the mean age of the individuals was 33.81 ± 14.09 (years) (Table 2).

**TABLE 2:** Descriptive characteristics of individuals (*n* = 479).

Demographic characteristics		*n*	%
Gender	Women	300	62.6
	Men	179	37.4
Marital status	Married	237	49.5
	Single	242	50.5
Education status	Literate	11	2.3
	Primary education	98	20.5
	Secondary education	164	34.2
	Higher education	178	37.2
	Postgraduate	28	5.8
Monthly income	Income less than expenses	124	25.9
	Income equal to expenses	317	66.2
	Income more than expenses	38	7.9
Chronic disease status	Yes	84	17.5
	No	395	82.5
Knowledge about epilepsy	Yes	330	68.9
	No	149	31.1
	$\bar{X}\pm SD\left(min-max\right)$		
Age (year)	33.81 ± 14.09 (18–79)		

Following the validation and reliability assessment of the measurement tools, a structural equation model was constructed to examine the relationship between the scales. The compatibility of the model was confirmed in line with the hypotheses, and the model fit indices demonstrated satisfactory results: *χ* ^2^/df = 1.857, RMSEA = 0.04, CFI = 0.98, GFI = 0.99, AGFI = 0.97, and IFI = 0.98. These indices indicated that the model met the desired criteria for fit (Karagöz 2019) (Table 3).

**TABLE 3:** Fit index values of the model.

Fit index	Research model	Normal value	Acceptable value
χ^2^/Sd	1.857	< 2	< 5
GFI	0.99	> 0.95	> 0.90
AGFI	0.97	> 0.95	> 0.85
IFI	0.98	> 0.95	> 0.90
CFI	0.98	> 0.95	> 0.90
RMSEA	0.04	< 0.05	< 0.08

As a result of the model:

**H_1_**. *Fatalistic tendency affects attitudes toward epilepsy patients*.

**FIGURE 3:**
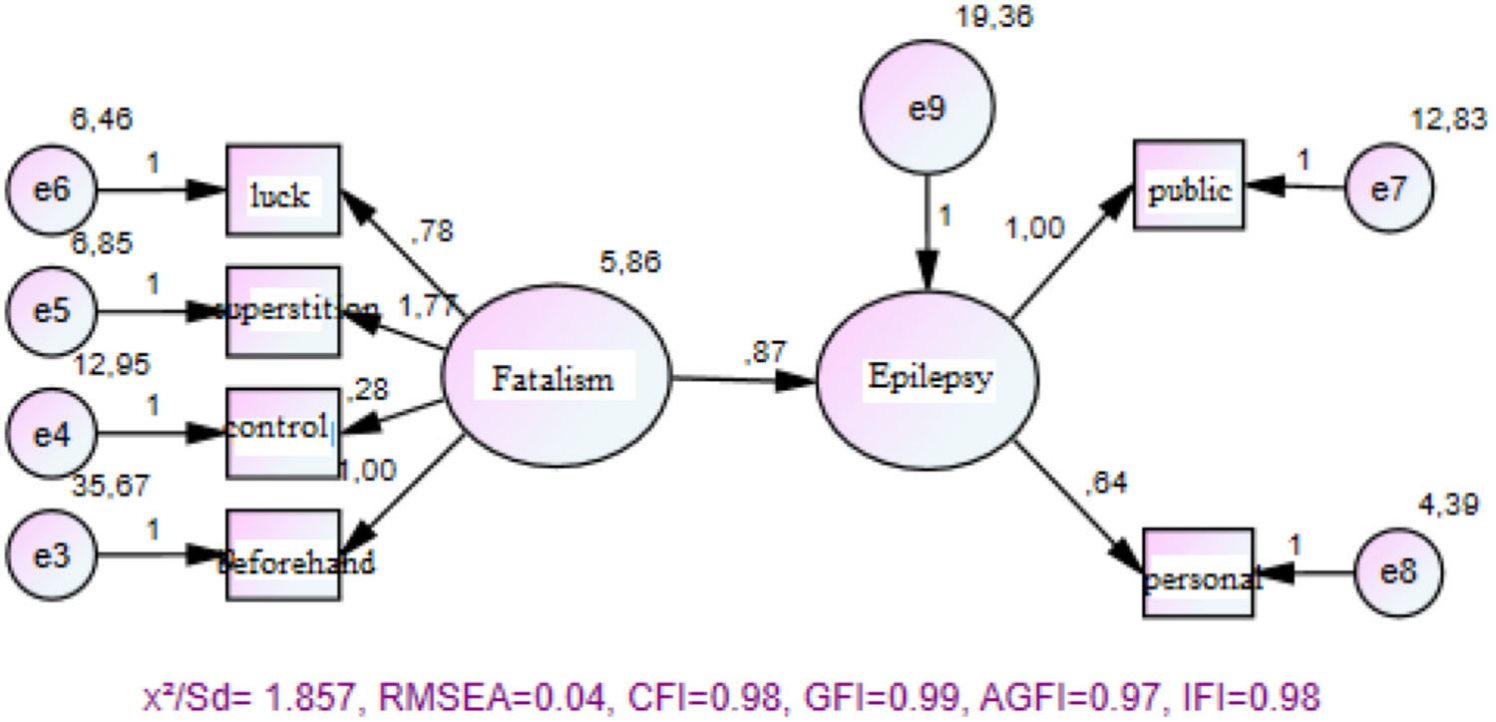
SEM diagram showing the relationship between fatalism tendency and attitudes toward epilepsy patients.

The hypothesis was confirmed (*p* < 0.05), and hypothesis H1 was accepted (Figure 3; Table 4).

**TABLE 4:** Investigation of the effect of fatalism tendency on attitudes toward epilepsy patients.

Independent variable	Dependent variable	*β*0	*β*1	S.E.	C.R	*p*
Fatalism tendency scale	Attitudes toward epilepsy scale	0.432	0.871	0.166	5.248	**0.001**

*Note: β*0 = standardized regression coefficient and *β*1 = nonstandardized regression coefficient.

In this study, Figures 1 and 2 show the confirmatory factor analysis of the scale and Figure 3 shows the SEM validation.

Confirmatory factor analysis of the FTS is shown in Figure 1. Confirmatory factor analysis indicated satisfactory fit indices: *χ* ^2^/df = 2.238, RMSEA = 0.05, CFI = 0.90, GFI = 0.91, AGFI = 0.89, and IFI = 0.90 confirming the structural integrity of the scale (Figure 1).

Confirmatory factor analysis of the PATE scale is shown in Figure 1. Confirmatory factor results of the analysis, the goodness of fit indices were *χ* ^2^/Sd = 3.777, RMSEA = 0.07, CFI = 0.93, GFI = 0.91, AGFI = 0.88, IFI = 0.93, and TLI = 0.91, and the structure of the scale was confirmed (Figure 2).

When the results of the SEM were analyzed, the compatibility of the model was confirmed in line with the hypotheses, and the model fit indices demonstrated satisfactory results: *χ* ^2^/df = 1.857, RMSEA = 0.04, CFI = 0.98, GFI = 0.99, AGFI = 0.97, and IFI = 0.98. These indices indicated that the model met the desired criteria for fit (Figure 3).

## 4 Discussion

In this part of the study, the findings on the effect of fatalistic tendencies on attitudes toward epilepsy patients are discussed in light of the literature.

Results in our study showed that fatalistic tendency influenced attitudes toward epilepsy patients (*p* < 0.05).

This scenario suggests that there is an association between an increase in individuals' fatalistic tendencies and a corresponding decline in their attitudes toward epilepsy patients, signifying a more negative perspective. Upon reviewing the literature pertinent to this phenomenon, it becomes apparent that this observed relationship aligns with existing research and contributes valuable insights to our understanding of the interplay between fatalism tendencies and attitudes toward epilepsy patients.

The greatest cause of limitation and discomfort for people with epilepsy is stigma. Stigmatization, which is unfortunately prevalent in many cultures, leads to the social isolation of people with epilepsy and their families (Tedrus, Pereira, and Zoppi 2018; Nelson and Robert 2019). Although there are campaigns to inform the public about epilepsy, there is still a lack of knowledge about interventions that can reduce stigma (Price et al. 2015; Herrmann et al. 2016).

Stigma reduction interventions can play a role in changing society's negative attitudes toward people with epilepsy and provide a supportive and positive environment for people with epilepsy (Kaddumukasa et al. 2018). Healthcare professionals have the greatest role to play in raising society's awareness of this issue (Alomar et al. 2020). To provide optimal health services, nurses and health technicians should be knowledgeable about epilepsy and have a positive attitude toward people with epilepsy (Dayapoğlu and Tan 2016; Higgins et al. 2018). In our study, it was demonstrated that a crucial issue that healthcare professionals should pay attention to is the fatalistic tendency of individuals.

People perceive their position as less dangerous when they feel that events are under their control. Those who reduce events to chance, fate, or the power of others are more likely to feel fear. For example, public attitudes toward mental illness have been found to be directly related to perception of patients as “dangerous” and “unpredictable”. Patients' unpredictable and unorthodox behavior, in short, their disruption of the existing order, creates fear in society. When an individual or society encounters a situation that frightens and disturbs them, they often choose to exclude and alienate them (Taşkın 2004).

It is conceptualized as an internal and external locus of control in terms of attributing the causes of an individual's behavior to internal or external factors. While individuals with an internal locus of control associate events with their behaviors, those with an external locus of control attribute them to factors such as luck and fate (Büyükgöze‐Kavas, Topkaya, and Gençoğlu 2014).

Given the negative attitudes toward epilepsy with fatalism, influenced with religious beliefs and cultural structures of society, psychological disorders may be associated with mystical powers and evil spirits or treated with fatalistic approaches (Sarıkoç et al. 2015).

The findings of the study conducted in northeastern Türkiye, examining attitudes toward epilepsy and health fatalism, revealed that individuals with elevated levels of health fatalism tended to exhibit more negative attitudes toward epilepsy (Kıyak et al. 2021).

## 5 Conclusion

This study found that fatalistic tendency affects attitudes toward epilepsy patients. As fatalism increases, so do negative attitudes toward epilepsy patients. Public education programs should be organized to minimize health fatalism and develop positive attitudes toward epilepsy patients. Longitudinal studies of attitudes toward epilepsy patients are recommended.

## 6 Limitations

The current study has several limitations. First, the data were obtained only from individuals who presented to a family health center. Hence, it is crucial to note that the outcomes might not be fully representative of the entire population of Türkiye, given the reliance on self‐report data and potential limitations associated with this sampling method. Secondly, it's essential to acknowledge that since the data were gathered through self‐report scales, the reliability of the information is contingent upon the accuracy of responses provided by the individuals participating in the study. The limitation of the study is that having individuals with different demographic characteristics in our study may affect the results of the study. SEM, which is a powerful statistical model, was used to overcome this limitation.

## Author Contributions

**Öznur Adadioğlu**: conceptualization, investigation, writing–original draft, methodology, validation, writing–review and editing, visualization, formal analysis, data curation, supervision, resources. **Ahmet Seven**: conceptualization, investigation, writing–original draft, methodology, data curation, validation. **Metin Yıldız**: conceptualization, validation, methodology, formal analysis.

## Conflicts of Interest

The authors declare no conflicts of interest.

### Peer Review

The peer review history for this article is available at https://publons.com/publon/10.1002/brb3.70160.

## Data Availability

The data that support the findings of this study are available from the corresponding author upon reasonable request.
